# Ultrasound Evaluation of Varicoceles: A Closed-Loop Audit of Compliance With European Society of Urogenital Radiology Guidelines

**DOI:** 10.7759/cureus.119453

**Published:** 2026-11-10

**Authors:** Hamza A Khan, Kethesparan Paramesparan

**Affiliations:** 1 Radiology, Basingstoke and North Hampshire Hospital, London, GBR; 2 Radiology, Croydon University Hospital, London, GBR

**Keywords:** audit, esur-spiwg, fertility, quality improvement projects, ultrasound (u/s), varicocele

## Abstract

Objective: To evaluate ultrasound reporting practices for varicoceles at a district general hospital in London against the European Society of Urogenital Radiology Scrotal and Penile Imaging Working Group (ESUR-SPIWG) standards and measure the impact of a structured reporting intervention.

Background: Varicoceles are common and may cause infertility and chronic scrotal discomfort. Ultrasound is the gold standard imaging modality for investigation. The ESUR-SPIWG guidelines, based on the Sarteschi grading system, mandate documentation of testicular volume, vein location and diameter (supine and erect), and duration of venous reflux. Previous audit data suggested poor compliance with these standards.

Methods: Retrospective closed-loop audit of ultrasound reports for 40 patients per cycle, investigated for varicoceles (Cycle 1: July 2023; Cycle 2: October 2024). Data were extracted from the Picture Archiving and Communication System (PACS) and CERNER (electronic health records). Three key reporting criteria were assessed per ESUR-SPIWG guidelines. A structured reporting template was introduced as an intervention between audit cycles.

Results: Full compliance with all three criteria improved from 0% in Cycle 1 to 22.5% (9/40 cases) in Cycle 2. Cases documenting at least one criterion increased from 47.5% (19/40) to 82.5% (33/40). While the intervention demonstrated improvement, substantial gaps in adherence remain.

Conclusions: The implementation of a structured reporting template showed measurable improvement in ESUR-SPIWG compliance; however, current practice still falls well short of the 100% target. This represents an audit cycle requiring continued quality improvement efforts. Further initiatives, including in-person education, intranet-accessible reporting pathways, and sustained reinforcement, are necessary to achieve guideline compliance and improve clinical decision-making for varicocele management.

## Introduction

Varicoceles, characterized by abnormal dilatation of the pampiniform venous plexus surrounding the spermatic cord, represent one of the most frequently encountered incidental findings on scrotal ultrasound imaging [1-5]. These lesions are commonly associated with male infertility [2-4], affecting approximately 40% of men with primary infertility and up to 80% with secondary infertility [2], and may result in chronic scrotal discomfort and potential progressive testicular dysfunction if not assessed accurately [6-8]. Ultrasound has become the gold standard imaging modality for the investigation, characterization, and grading of varicoceles, offering excellent spatial resolution, real-time imaging capability [2,5], and the ability to assess hemodynamic parameters through Doppler assessment during physiologic maneuvers such as the Valsalva maneuver.

The European Society of Urogenital Radiology Scrotal and Penile Imaging Working Group (ESUR-SPIWG) has established comprehensive standardized reporting guidelines for varicocele assessment [1], based on the Sarteschi grading system, which incorporates five grades of varicocele severity [6,7]. These guidelines mandate documentation of several essential parameters: measurement of testicular volume using Lambert's formula (length × width × height × 0.71), which is widely used as the most accurate way to measure true testicular size [1,6], precise localization of the varicocele (inguinal, supratesticular, or peritesticular), measurement of the largest vein diameter in both supine and erect positions, and critical documentation of the location and duration of venous reflux during the Valsalva maneuver in both body positions. The duration of reflux is particularly important clinically, as reflux lasting longer than two seconds correlates with improved prognosis for post-operative enhancement of semen quality following surgical intervention [3,7].

Despite the existence of these international guidelines and their clinical importance, compliance across institutions remains highly variable, potentially compromising diagnostic accuracy, clinical decision-making, and patient management pathways [1,2].

## Materials and methods

This closed-loop audit aimed to evaluate current ultrasound reporting practices for varicoceles at NHS District General Hospital in South London, against established ESUR-SPIWG [1] and RCR (Royal College of Radiologists) [9] standards, and to measure the impact of a structured reporting template intervention on improving guideline compliance and reporting quality.

A two-cycle retrospective audit was conducted on ultrasound reports from patients investigated specifically for varicoceles. Cycle 1 included 40 consecutive patients examined in July 2023, with results initially presented in April 2024, which revealed baseline findings of 0% compliance with ESUR-SPIWG reporting guidelines [1]. Following formal presentation and identification of this significant quality gap, a structured reporting template was collaboratively developed by radiologists and sonographers to standardize and streamline the reporting process. Cycle 2 audit was conducted in October 2024 using another set of 40 patient reports, with the same inclusion criteria applied, to measure the effectiveness of the intervention. All ultrasound reports were retrieved from both the Picture Archiving and Communication System (PACS) [10]** **and the hospital Electronic Health Record (CERNER).

Inclusion criteria were defined as any ultrasound examinations of the testes with specific documentation of varicocele evaluation and findings in July 2023 (Cycle 1) and October 2024 (Cycle 2). Exclusion criteria included ultrasound examinations performed without mention of varicocele findings and cases where access to complete reports was unavailable.

Three key reporting criteria aligned with ESUR-SPIWG recommendations [1] were established as the audit standards: (1) routine measurement of testicular volume for both testes using Lambert's formula [6]; (2) documentation of varicocele location (inguinal, supratesticular, or peritesticular) and documentation of the largest vein diameter in both supine and erect positions; and (3) documentation of the location of venous reflux (at the inguinal, supratesticular, or peritesticular level) and the duration of this reflux in both supine and erect positions during Valsalva maneuver [1]. Full compliance was defined as documentation meeting all three criteria; partial compliance as documentation meeting one to two of the three criteria; and finally, any compliance was defined as at least one of the criteria being included. The reports were assessed for quality and against the criteria independently by two doctors (a Consultant in Clinical Radiology and a Senior House Officer).

Intervention

Based on the findings of Cycle 1, the need for interventions to improve adherence to the ESUR-SPIWG criteria was apparent [1,11] and, in accordance with audit methodology principles, a structured reporting template was specifically designed to facilitate systematic and complete documentation of all ESUR-SPIWG criteria [1,12]. The template incorporated explicit sections for each reporting requirement [12], included prompts for measurement methodology with use of Lambert's formula [6], position-dependent assessment (supine versus erect), and structured fields for documenting reflux location and duration. The template was disseminated to all five sonographers and five radiologists involved in scrotal ultrasound reporting through departmental meetings and written guidance.

Each cycle of this closed-loop audit was registered with Croydon University Hospital's audit office (registration numbers: 2024/039 and 2024/077, respectively) after official approval from the departmental lead for Clinical Radiology. Ethics approval was not required as there was no direct human involvement.

## Results

Cycle 1 audit results demonstrated non-compliance with established ESUR-SPIWG guidelines [1] across all measured parameters (Table 1). None of the reports demonstrated full compliance with all three key reporting criteria, representing a complete absence of standardized reporting practice at the institution. This finding was particularly concerning given the well-established clinical importance of these parameters in varicocele management [1,7,8]. Partial compliance, defined as one to two of the criteria included, was observed in 47.5% (19/40) of cases, indicating that while some reports documented at least one of the required parameters, systematic and comprehensive documentation was decidedly not standard practice. Of particular note, when individual criteria were analyzed, testicular volume measurement was documented in only 12.5% (5/40) of reports, vein diameter and location in supine/erect positions in 22.5% (9/40) of cases, and reflux duration documentation in 17.5% (7/40) of cases. This granular analysis suggests widespread knowledge gaps regarding ESUR-SPIWG requirements [1] among the reporting team. This finding highlighted a significant quality gap and justified the implementation of formal quality improvement interventions (Table 1).

**Table 1:** ESUR-SPIWG reporting compliance, Cycle 1 vs. Cycle 2 (n = 40 each) ESUR-SPIWG: European Society of Urogenital Radiology Scrotal and Penile Imaging Working Group

Variable	Cycle 1 (n = 40)	Cycle 2 (n = 40)	Test used	Test statistic	p-value
Full compliance (all 3 criteria)	0 (0%)	9 (22.5%)	Fisher’s exact	-	0.0024
Partial compliance (1-2 of 3 criteria)	19 (47.5%)	24 (60%)	Chi-square	1.26	0.26
Any compliance (≥1 of 3 criteria)	19 (47.5%)	33 (82.5%)	Chi-square	10.77	0.001
Testicular volume documented	5 (12.5%)	19 (47.5%)	Chi-square	11.67	0.0006
Vein diameter and location (supine/erect)	9 (22.5%)	22 (55%)	Chi-square	8.90	0.003
Reflux duration documented	7 (17.5%)	24 (60%)	Chi-square	15.22	0.0001

Following implementation of the structured reporting template and associated educational initiatives [11], Cycle 2 results demonstrated some improvement across both compliance metrics, though gaps remained (Table 1). Full compliance with all three ESUR-SPIWG reporting criteria [1] increased from 0% to 22.5% (9/40 cases), representing an absolute improvement. While this represents a meaningful advancement that demonstrates the effectiveness of the intervention, it also demonstrates that 77.5% (31/40) of reports still failed to meet the complete standard. Analysis of individual criteria in Cycle 2 revealed: testicular volume measurement improved to 47.5% of reports (19/40), vein diameter and location documentation increased to 55% (22/40), and reflux duration documentation reached 60% (24/40). These improvements suggest differential adoption of the template components, with certain parameters being more consistently implemented than others (Table 1).

Partial compliance - defined as documentation of one to two of the criteria - improved from 47.5% (19/40) to 60% (24/40) cases, an absolute increase of 12.5% (5/40) cases. This metric indicates that awareness of the reporting requirements has increased to an extent, albeit not significantly, and the majority of reports now include at least some of the guideline-mandated information (Table 1). Results also reveal that 17.5% (7/40) of varicocele studies lack any systematic documentation aligned with international standards [1].

The pattern of improvement across metrics suggests that while sonographers and radiologists are becoming increasingly aware of and compliant with reporting requirements, systematic adherence to all criteria simultaneously remains incomplete. Several factors may explain the partial compliance plateau: (1) time constraints in busy clinical practice; (2) incomplete template integration into all reporting workflows; (3) possible resistance to standardized reporting among certain staff members; and (4) technical limitations in documenting all parameters in every patient, e.g., inability to achieve adequate erect position imaging in some cases [11,13]. These results suggest that the structured template intervention was effective in raising awareness and improving documentation practices [11,12]. Sustainability of these improvements will require ongoing departmental commitment and periodic re-audit cycles.

## Discussion

Incomplete documentation of varicocele characteristics can substantially and negatively impact clinical decision-making and patient management pathways [5,11]. Urologists rely heavily on precise and complete documentation provided by radiologists and sonographers to accurately stratify disease severity, determine individual candidacy for surgical intervention, predict likely surgical outcomes, and estimate the probability of post-operative improvement in semen quality parameters [5,7]. The quality of radiologic reporting directly influences urologic decision-making, particularly in determining whether a varicocele is of subclinical importance or truly relevant: a patient with inadequately characterized varicoceles may be either inappropriately offered surgery despite mild disease, or conversely, denied beneficial intervention due to underestimation of disease severity [5].

Varicocele grading according to the Sarteschi system, as mandated by ESUR-SPIWG guidelines [1], directly influences clinical management decisions and prognostication. Higher grades of varicoceles-particularly those demonstrating retrograde venous reflux lasting longer than two seconds-are associated with significantly improved outcomes following corrective surgical procedures, including objective improvement in semen parameters and enhanced fertility potential [3,5,7]. Conversely, lower-grade and subclinical varicoceles may not warrant surgical intervention in asymptomatic men, particularly in those with normal semen analyses [2,7]. Without precise documentation of reflux duration, reflux location, and vein diameter, urologists cannot reliably assign Sarteschi grades and must either request additional imaging studies (increasing costs and patient burden) or make management decisions based on incomplete information [1,7].

The consequences of suboptimal reporting extend beyond individual patient care [11]. Incomplete varicocele characterization has direct implications for research and quality assurance initiatives, potentially distorting outcome analyses and leading to erroneous conclusions about the effectiveness of varicocele interventions [3]. Furthermore, standardized reporting facilitates communication between different institutions, enables meaningful comparison of outcomes across centers, and supports the development of evidence-based clinical guidelines [13].

The improvement in compliance demonstrated in this audit cycle - while modest at 22.5% (9/40 cases) full compliance - represents meaningful progress toward better patient care. The increase in partial compliance to 60% (24/40) suggests that awareness-raising efforts are working and that many practitioners are beginning to integrate guideline-compliant documentation into their practice. However, the persistence of 77.5% (31/40 cases) non-compliance with complete guidelines underscores the need for sustained quality improvement efforts [11]. Each percentage point improvement in compliance represents dozens of patient cases that will receive more appropriate characterization, potentially leading to optimized clinical decision-making, improved patient outcomes, and enhanced semen quality improvements following surgical intervention for appropriately selected candidates [1,11].

Limitations

This study has several important limitations that warrant acknowledgment. Firstly, as a single-institution audit, the findings may not be generalizable to other NHS trusts, private practice settings, or institutions with differing departmental structures and staffing models [13]. Second, the retrospective design is inherently prone to documentation bias; incomplete reporting of ESUR-SPIWG parameters may reflect gaps in documentation practices rather than failures in imaging technique or protocol adherence [14]. The absence of a contemporaneous control group limits the ability to isolate the specific impact of the structured template intervention from other concurrent factors that may have influenced compliance, such as broader institutional awareness campaigns, staffing changes, or external guidance dissemination during the study period [13].

The modest sample size of 40 cases per cycle, while appropriate for a departmental audit, results in relatively wide confidence intervals and limits statistical power [15]. The interval between Cycle 1 and Cycle 2 introduces potential confounding variables, including staff turnover, equipment changes, and evolving practice patterns [14].

The bundling of multiple interventions (structured template implementation, departmental educational meetings, and awareness-raising activities) precludes definitive determination of which component(s) drove observed improvements [11]. Compliance assessment was performed retrospectively from written reports without inter-observer or intra-observer reliability testing, potentially introducing assessment bias [14]. Critically, this study measured documentation compliance rather than clinical outcomes; whether improved radiologic reporting translates to enhanced urologic decision-making, optimized patient management, or improved post-operative fertility outcomes remains unestablished and warrants prospective investigation through auditing.

Technical limitations in imaging acquisition - such as inability to achieve adequate erect-position imaging in some patients due to physical constraints or discomfort - could not be assessed from report analysis alone and may account for some documented gaps in position-dependent parameters [16]. The durability and sustainability of compliance improvements beyond the immediate post-intervention period remain unknown, as formal follow-up assessment was not conducted beyond the October 2024 audit cycle. Finally, this study did not take into account how many times the template was used. This would be useful information to assess in further cycles.

## Conclusions

This complete loop audit cycle demonstrates that implementation of a structured reporting template resulted in measurable improvement in ESUR-SPIWG guideline compliance for varicocele ultrasound reporting, increasing full compliance. While this represents genuine progress, substantial gaps persist, with the majority of reports still failing to achieve comprehensive guideline adherence. The findings underscore the importance of systematic quality improvement initiatives in standardizing clinical practice. Recommended next steps include sustained reinforcement through continuing departmental education, creation of easily accessible intranet-based reporting pathways, and a formal third-cycle audit scheduled post-intervention to assess sustainability and guide further quality enhancement initiatives. Ultimately, achieving the full compliance target should improve diagnostic accuracy, facilitate optimal clinical decision-making, and enhance patient outcomes in varicocele management.
